# Preoperative Risk Factors of Persistent Pain following Total Knee Arthroplasty

**DOI:** 10.1155/2022/4958089

**Published:** 2022-12-15

**Authors:** Paweł Chodór, Jacek Kruczyński

**Affiliations:** Department of General Orthopedics, Orthopedic Oncology and Traumatology, University of Medical Sciences, Poznań, Poland

## Abstract

**Background:**

Despite good results of total knee arthroplasty (TKA) as a treatment of idiopathic osteoarthritis (OA) of the knee, significant number of patients (16-33%) complain of persistent pain of unknown origin. This phenomenon is the major cause of patient's dissatisfaction. It has been theorized that certain preoperative factors may increase the risk of persistent pain; hence, their identification should enable proper preoperative education and development of realistic expectations regarding results of TKA. This study is aimed at identifying the preoperative chronic pain predictors in patients undergoing TKA.

**Methods:**

In this prospective cohort study, patients scheduled for TKA were examined one day prior to surgery. Demographics, comorbidities, pressure pain thresholds, pain intensity and duration, radiographic OA grade, and range of motion were recorded. Questionnaires such as Beck Depression Inventory (BDI) and Knee Injury and Osteoarthritis Outcome Score (KOOS) were collected. Study cohort was evaluated approximately 6 months following surgery. Patients were assigned to group A if they had no pain and to group B if they complained of any pain. Collected data was analyzed by biostatistician.

**Results:**

64 patients were included in final analysis, 49 (76,6%) females and 15 (23,4%) males. Mean age was 67,6 yrs (48-84, ±7,42). Group A consisted of 21 patients (33%) while group B consisted of 43 patients (67%). There were no statistically significant differences regarding preoperative factors except for duration of preoperative pain, which was shorter in group A (36 (12-180) vs. 72 (24-180), *p* = 0,011). Every 12 months of preoperative pain were found to increase risk of persistent pain by 1,27 (*p* = 0,009).

**Conclusions:**

Preoperative duration of pain is a risk factor for chronic pain following TKA. Therefore, patients should be operated on as soon as indications arise. Should the surgical treatment of knee arthritis be postponed, intensive and individualized pain management is highly recommended.

## 1. Introduction

Knee osteoarthritis (OA) is one of the leading and highly prevalent causes of pain and disability in developed populations [1]. In population over 45 years old, radiographic signs of OA are present in approx. 30% of patients, with over half of them being symptomatic [2]. Symptomatic knee osteoarthritis is more frequently observed in females than in males [3]; moreover, pain experienced by females is more severe. Knee osteoarthritis in associated with comorbidities, which partially stem from lack of physical activity, obesity, and medication toxicity [4].

Treatment of symptomatic knee osteoarthritis may be nonoperative or operative. It is recommended to begin with conservative treatment, which consists of pharmacotherapy, physical therapy, bracing, etc. [5]. Should conservative treatment prove ineffective, total knee arthroplasty (TKA) is considered a treatment of choice in severe, symptomatic OA of the knee [6]. Based on data from arthroplasty registries, hundreds of thousands knee arthroplasties are being performed annually worldwide. Since 1975, over 2.5 million primary total knee arthroplasties were performed in Europe according to Lübbeke et al. [7].

Despite high cost-effectiveness, total knee arthroplasty may render suboptimal results, such as chronic knee pain, described as pain lasting 3-6 months after surgery. This may occur in 16-33% of patients undergoing total knee arthroplasty (TKA) [8]. Given the high number of procedures worldwide, chronic pain after TKA is a major issue affecting thousands of patients.

Causes of painful knee after TKA have been widely investigated, and numerous biomechanical factors were identified [9, 10]. These mainly include malposition of the implant (valgus/varus and malrotation), lack of proper ligamentous balance, patellar maltracking, or aseptic/septic loosening. Nevertheless, in substantial number of patients with chronic pain, those problems are not present. Recently, it has been theorized that mechanisms of neuromodulation, i.e., central sensitization, may contribute to unclear chronic pain following TKA [11, 12]. Symptoms of central sensitization like hyperalgesia and allodynia may be expressed in lowered pressure pain thresholds (PPTs) [13]. Moreover, patients' psychological status (depression and anxiety) may also have impact on development of chronic pain [14].

It has been found that unsatisfactory pain relief is one of the major factors influencing patient's satisfaction after TKA [15]. Therefore, the aim of this study was to identify preoperative risk factors of chronic pain following TKA, which may allow better education and developing realistic expectations in patients scheduled for TKA.

## 2. Material and Methods

In this prospective cohort study, participants were recruited consecutively among patients scheduled for TKA according to inclusion criteria. Surgical treatment (TKA) was performed in high-volume joint replacement institution by experienced orthopedic surgeons. After discharge from the hospital, participants were followed for at least 6 months. After half a year, patients were assigned to study groups: group A with no pain and group B with any pain in the operated knee.

From March to December 2016, patients admitted to the orthopedic department and scheduled for total knee arthroplasty were recruited for the study. Inclusion criteria were as follows: idiopathic, severe, and symptomatic osteoarthritis of the knee and anticipated cruciate retaining implant. Exclusion criteria included lack of informed consent, symptomatic osteoarthritis of the ipsilateral hip, sacroiliac joint, and severe sciatica.

Patients were assessed preoperatively by trained orthopedic surgeons according to the standardized protocol, which comprised of detailed anamnesis (demographics, comorbidities, pain duration, etc.) and physical examination of the knee (including range of motion measurement with the goniometer). Pressure pain threshold (PPT) assessments in the medial joint line of the knee and over extensor carpi radialis brevis on the contralateral forearm were performed using pressure algometer (Force Dial Algometer, Wagner Instruments). Varus/valgus deformity and Ahlback OA grade were evaluated on the preoperative standing AP X-rays. Participants were given Beck Depression Inventory (BDI), Knee Injury and Osteoarthritis Outcome Score (KOOS), and a Visual Analogue Scale (VAS).

Pressure algometry has been proven to provide good test-retest reliability in assessing pain in patients with OA [16]. Examination may be performed using wide variety of manual or computerized algometers [17, 18]. Manual Wagner Instruments FPK Algometer was previously successfully implemented in pressure pain threshold assessment [19].

Beck Depression Inventory is a screening test for depression, which consists of 21 questions with answers ranging from 0 to 3. If the patient scores above 11 points, the test is considered positive. It has been used in diagnosing intensity of depression in numerous fields (chronic pain, rheumatology, cardiac patients, etc.) [20].

Knee Injury and Osteoarthritis Outcome Score (KOOS) was developed as an extension of WOMAC Osteoarthritis Index and has been proven to be useful tool for evaluation of degenerative knee joint [21].

Visual Analogue Scale is a reliable, simple tool for pain intensity assessment [22, 23]. Patients are asked to mark pain intensity on a 10 cm ruler (many variants exist in literature, with pictograms or colours).

Total knee arthroplasty was performed according to a standard surgical technique. Postoperative X-rays were taken in the first postoperative day. Radiological parameters, such as medial distal femoral angle (MDFA), medial proximal tibial angle (MPTA), implant size (notching and overhang), sagittal position of the femoral implant (flexion, extension, or neutral), and tibial slope and posteriori condyle offset ratio (PCO ratio), were assessed [24]. Patients were discharged from hospital 5-7 days after surgery.

Participants were assessed in an outpatient setting after 6 months following surgery. Standing AP and lateral X-ray of both knees were obtained to exclude loosening, periprosthetic fracture, catastrophic polyethylene wear, etc. PPTs were measured and BDI and KOOS questionnaires along with VAS were collected. Patients were asked whether they are satisfied with an outcome of the surgery and whether pain significantly limits their daily activity. Moreover, physical examination of the operated knee joint was performed, and range of motion and presence of any abnormal laxity were recorded. Then, patients were divided in two groups. Group A consisted of patients with no pain in the operated knee, whereas group B consisted of those who suffered from a painful knee.

Recruitment of participants was discontinued after reaching 69 participants which corresponded with previously published study by Lundblad et al. and was considered sufficient by author [25].

The obtained data was analyzed by a biostatistician using StatSoft, Inc. (2014). STATISTICA version 12 was used with significance level set for *p* = 0, 05. For a parametrical variable with a normal distribution, Student's *t* and Cochrane-Cox tests were used. The Mann–Whitney *U* test was used to compare nonparametrical data. For dichotomic, nonparametrical variables, chi^2^ and Fisher exact tests were used. Correlations were tested with Spearman's rank correlation coefficient. Logistic regression was performed for statistically significant variables.

All procedures were in accordance with the ethical standards of the responsible committee on human experimentation.

## 3. Results

In the final analysis, 64 patients were included. Five were excluded from the study: one patient was diagnosed with early periprosthetic join infection, there was one case of metal hypersensitivity, and three patients were lost to follow-up. There were 76,6% (49 pts) of females and 23,4% of males (15 pts) in study population, and mean age was 67,6 yrs (48-84, ±7,42). Mean follow-up time was 231,6 days (181-318, ±34,3). 67,2% of patients were diagnosed with arterial hypertension, and 26,6% suffered from circulatory diseases other than hypertension, whereas 18% were diagnosed with diabetes mellitus. 14% of patients had hypothyroidism and 1,5% suffered from epilepsy. There were no cases of ischemic stroke or depression in study cohort (Table 1).

After the follow-up, there were two groups of patients: group A (pain-free) comprised of 21 (33%) patients and group B (any pain), which consisted of 43 (67%) patients. There were no statistically significant differences in terms of age (69, 9 ± 6, 2 vs. 66, 5 ± 7, 8, *p* = NS) or gender (females 76% vs. 77%, *p* = NS; males 24% vs. 23%, *p* = NS).

There were no statistically significant differences between groups A and B in respect of arterial hypertension (62% vs. 69,8%, *p* = NS), circulatory diseases (28,6% vs. 25,6%, *p* = NS), diabetes mellitus (14,3% vs. 21%, *p* = NS), hypothyroidism (14,3% vs. 14%, *p* = NS), and epilepsy (0% vs. 2,3%, *p* = NS). In terms of preoperative VAS score (7 (4,5-9,5) vs. 7 (3-10), *p* = NS), as well as total WOMAC score (57 (33-80) vs. 58 (9-83), *p* = NS), no significant differences were observed. Duration of preoperative pain was significantly shorter in group A than in group B (36 mo. (12-180) vs. 72 mo. (24-180), *p* = 0, 011294). Preoperative flexion (101,2 ± 13, 3 deg vs. 105 ± 13, 2 deg, *p* = NS) and flexion contracture (10 deg (0-20) vs. 10 deg (0-30), *p* = NS), as well as Ahlback grade (3 (1-5) vs. 2 (1-5), *p* = NS), did not differ between study groups.

In KOOS subscale such as pain (44,0 (19,0-67,0) vs. 42,0 (14,0-86,0), *p* = NS), other symptoms (39,0 (18,0-75,0) vs. 36,0 (4,0-100,0), *p* = NS), activities of daily life (41,0 (9,0-72,0) vs. 40,0 (12,0-90,0), *p* = NS), and quality of life (20, 8 ± 14, 8 vs. 25, 7 ± 13, 5, *p* = NS), patients from groups A and B had similar results.

No correlation was found between BDI score and postoperative pain intensity. No statistically significant differences between groups A and B were observed in terms of PPTs in the joint line (2,1 (1,0-5,5) vs. 2,5 (1,0-10,0), *p* = NS) and on the contralateral forearm (3,2 (1,0-8,0) vs. 3,2 (1,5-7,2), *p* = NS). Study groups were fairly similar in respect of radiological parameters of the operated knee (Table 2.

Six months after the surgery, patients were examined in terms of range of motion, and no differences in flexion (100 deg (80-120) vs. 110 deg (90-120), *p* = NS) and flexion contracture (0 deg (0-20) vs. 0 deg (0-15), *p* = NS) were observed. No signs of loosening or periprosthetic fracture were found. In respect of KOOS subscales such as pain (88, 1 ± 7, 5 vs. 63, 2 ± 19, 0, *p* < 0, 0001), other symptoms (79, 7 ± 11, 2 vs. 58, 8 ± 16, 8, *p* < 0, 0001), activities of daily life (84, 9 ± 11, 6 vs. 61, 2 ± 18, 5, *p* < 0, 0001), and quality of life (68, 3 ± 16, 1 vs. 46, 8 ± 17, 4, *p* < 0, 0001), significant differences were observed, which was also seen in WOMAC total score (14, 7 ± 9, 0 vs. 35, 4 ± 16, 0, *p* < 0, 0001). PPTs were significantly higher in group A compared to group B both in the joint line (4,6 (2,5-8,0) vs. 3,0 (1,0-10,0), *p* = 0, 000277) and on the contralateral forearm (5,5 (3,2–9,0) vs. 3,6 (1,0–10,0), *p* = 0, 000675). None of the patients in group A described pain as severely limiting daily life compared to 25,6% of patients in group B confirming such limitations (*p* = 0, 0149). Satisfaction rate in group A was 95,2% compared to 67,4% in group B (*p* = 0, 03151). Logistic regression was performed for duration of preoperative pain and odds ratio was calculated. 1,27-fold increase in prevalence of chronic postsurgical pain with every 12 months of preoperative pain duration was found (*p* = 0, 008779) (Table 3).

## 4. Discussion

Chronic pain following TKA has been widely investigated in recent decade in an attempt to estimate its prevalence and possible causes. Previously implemented methods of TKA efficacy assessment supported the statement of it being highly successful treatment of severe knee OA [6]. In terms of implant, survival results are indeed satisfactory; however, considerable number of patients continue to experience pain following TKA [8]. Percentage of patients suffering from chronic pain varies, ranging from 16% to 33% [8]. This phenomenon has been explored in several studies, in which authors implemented various methods of pain assessment, study designs, and follow-up periods. Liu et al. found 53% of patients suffering from chronic pain, although response rate was relatively low (32%), while Wylde et al. observed chronic pain in 44% of patients after TKA [26, 27]. On the other hand, Baker et al. reported 19,8% of patients complaining of pain one year following TKA [28]. In our study, only 32% of patients had no pain in the operated knee 6 months after surgery. This may be attributed to relatively short follow-up. According to Heiberg et al., while most significant pain relief is seen in 3-6 months after surgery, slow improvement may also be observed up to one year following TKA [29]. In our study, patients with mild pain (VAS 1-4) constituted 44% (19 pts) of group B. These patients might have been tested during period of slow recovery which would have led to complete recovery.

No evidence of gender or age impact on CPSP was found, which is consistent with some previous studies [14, 25, 30]. However, some authors stated that younger age was related with higher prevalence of CPSP [31, 32]. Female gender was found to be a risk factor of CPSP by Puolakka et al. and Liu et al. [33, 34]. Neither comorbidities nor depression has proven to influence CPSP, although the latter has been shown to be a risk factor in some studies [14, 27, 35]. On the other hand, Sambamoorthi et al. reported depression to be a common comorbidity among patients with OA [36]. However, the rate of depression varies over a wide range (4,1%-61,3%) depending on the diagnostic criteria. In our study, no cases of depression were found; moreover, no correlation between BDI score and pain intensity was observed.

Despite some evidence suggesting predictive value, PPTs have not been able to reproduce those results. For instance, Wright et al. and Wylde et al. observed lower preoperative pressure pain thresholds in patients experiencing pain 12-18 months following TKA [11, 12]. Interestingly, in a study with larger cohort, no such correlation was reported [37]. Moreover, according to Petersen et al., static pain testing methods have no predictive value in terms of CPSP following TKA [38]. More advanced, dynamic methods assessing temporal summation of pain and conditioned pain modulation may be helpful in predicting CPSP [39].

Preoperative pain duration has been proven to impact prevalence of CPSP after TKA. According to Puolakka et al., with every year of pain in preoperative period increases risk of CPSP 2,99-fold [33]. In our study, this correlation has been confirmed, although risk increased 1,27-fold per every 12 months of preoperative pain.

Data on the impact of preoperative pain intensity on prevalence of CPSP is inconsistent. In our study, no clear link with CPSP was observed, while other authors confirmed such relationship. Lundblad et al. and Lindberg et al. stated that intense preoperative pain deteriorates results of TKA in terms of chronic pain [25, 40]. On the other hand, in the study from Singh et al., preoperative pain intensity increased risk of CPSP only in revision procedures [32]. No relationship between preoperative pain intensity and CPSP was found by Forsythe et al. [41]. Nevertheless, in meta-analysis by Lewis et al., preoperative pain intensity was considered to be a risk factor of CPSP [14].

The major limitation of this study is a relatively small number of patients included which was attributed to large exclusion rate at the time of recruitment (patients with posttraumatic OA, OA secondary to rheumatoid arthritis, revision surgeries after HTO or UKA, etc.). Short follow-up may be perceived as a limitation, although 6 months is sufficient to meet the criteria of persistent pain.

In our opinion, there are numerous pitfalls in assessment of chronic pain after TKA. Previous studies focused on pain testing and questionnaires, without screening patients for apparent causes of pain (loosening, instability, component malposition, PJI, etc. [9]). Moreover, indications for TKA were nonuniform or unknown (rheumatoid arthritis, posttraumatic arthritis, osteoarthritis, etc.), while TKA in posttraumatic arthritis may be less successful than TKA in primary OA in terms of pain [42]. Hardly any information of preoperative ROM or degree of valgus/varus deformity was given, which may be confusing because severe valgus deformity is more challenging in TKA and results may be relatively worse than in varus knees [43, 44]. We believe that this study shows prevalence of truly unclear pain in patients 6 months after primary TKA and identifies its predictor, which is long lasting, poorly controlled preoperative pain.

## 5. Conclusions

Preoperative duration of pain is a risk factor for CPSP following total knee arthroplasty; thus, patients should be operated on as soon as indications arise and conservative treatment proves ineffective. Should the surgical treatment of knee arthritis be postponed, intensive and individualized pain management is highly recommended.

## Figures and Tables

**Table 1:** Demographics and comorbidities of the study population (with range and standard deviation in brackets where applicable).

Age (yrs)	67,6 (48-84, ±7,42)
Female	76,7%
Male	23,3%
Follow-up (days)	231,6 (181-318, ±34,3)
Hypertension	67,2%
Circulatory diseases (other than hypertension)	26,6%
Diabetes mellitus	18%
Hypothyroidism	14%
Epilepsy	1,5%
Ischemic stroke/TIA	0
Depression	0

**Table 2:** Comparative analysis of preoperative parameters (in brackets range and standard deviation where applicable). kgf: kilogram-force.

	Group A	Group B	*p*
Age (yrs)	69, 9 ± 6, 2	66, 5 ± 7, 8	NS
Female	76%	77%	NS
Male	24%	23%	NS
Hypertension	62%	69,8%	NS
Circulatory diseases	28,6%	25,6%	NS
Diabetes mellitus	14,3%	21%	NS
Hypothyroidism	14,3%	14%	NS
Epilepsy	0%	2,3%	NS
VAS	7 (4,5-9,5)	7 (3-10)	NS
WOMAC	57 (33-80)	58 (9-83)	NS
KOOS pain	44,0 (19,0-67,0)	42,0 (14,0-86,0)	NS
KOOS other symptoms	39,0 (18,0-75,0)	36,0 (4,0-100,0)	NS
KOOS activities of daily life	41,0 (9,0-72,0)	40,0 (12,0-90,0)	NS
KOOS quality of life	20, 8 ± 14, 8	25, 7 ± 13, 5	NS
Ahlback grade	3 (1-5)	2 (1-5)	NS
PPTs knee (kgf)	2,1 (1,0-5,5)	2,5 (1,0-10,0)	NS
PPTs forearm (kgf)	3,2 (1,0-8,0)	3,2 (1,5-7,2)	NS
Flexion (°)	101,2 ± 13, 3	105 ± 13, 2	NS
Flexion contracture (°)	10 (0-20)	10 (0-30)	NS
Pain duration (mo.)	36 (12-180)	72 (24-180)	0,011294

**Table 3:** Comparative analysis of postoperative parameters (in brackets range and standard deviation where applicable). kgf: kilogram-force.

	Group A	Group B	*p*
Flexion (°)	100 (80-120)	110 (90-120)	NS
Flexion contracture (°)	0 (0-20)	0 (0-15)	NS
KOOS pain	88, 1 ± 7, 5	63, 2 ± 19, 0	<0,0001
KOOS other symptoms	79, 7 ± 11, 2	58, 8 ± 16, 8	<0,0001
KOOS activities of daily life	84, 9 ± 11, 6	61, 2 ± 18, 5	<0,0001
KOOS quality of life	68, 3 ± 16, 1	46, 8 ± 17, 4	<0,0001
WOMAC	14, 7 ± 9, 0	35, 4 ± 16, 0	<0,0001
PPTs knee (kgf)	4,6 (2,5-8,0)	3,0 (1,0-10,0)	0,000277
PPTs forearm (kgf)	5,5 (3,2–9,0)	3,6 (1,0–10,0)	0,000675
Severe limitation of daily life	0%	25,6%	0,0149
Satisfaction	95,2%	67,4%	0,03151

## Data Availability

Database is available upon request (via corresponding author's email).
